# Clinical Remarks on Certain Diseases of the Eye, and on Miscellaneous Subjects, Medical and Surgical, &c.

**Published:** 1844-01-01

**Authors:** 


### Clinical Remarks on certain Diseases of the Eye, and on Miscellaneous Subjects, Medical and Surgical, &c. By *John Charles Hall*, M.D. &c. London, John Churchill, 1843, pp. 228.

When we contrast the present state of ophthalmological knowledge with the comparative obscurity which existed in this interesting department of the science of medicine at so recent a period as the commencement of the present century, we must admit that a considerable advance has taken place both in theory and practice. The obscurity and mystery of empiricism have given place to the clearness and precision of genuine knowledge—this desirable change being effected by unprejudiced observation, patient industry, common sense, and a strict adherence to inductive reasoning. It must be admitted however, also, that much still remains to be done in order to perfect what has been so happily begun. We want a faithful description of the varieties and modifications of such diseases as are at present known, with the correspondent modifications found by experience to be most suitable in their treatment. Such information could only be furnished by the unprejudiced collection of facts for a series of years, and then cautiously reasoning on this solid basis. A strictly clinical work on the diseases of the eye, composed in the manner and with the intentions we have described, would therefore be a valuable boon to the profession, notwithstanding that we already possess several books which treat at large of these pathological affections, and are most methodically and minutely subdivided.

Dr. Hall tells us, in the preface to his work, that " the remarks offered in each chapter are the result of experience at the bedside of the patient." With an assurance at starting so consolatory, we shall endeavour to ascertain how far the contents correspond with the book of Nature, which Dr. H., in several parts of his volume, states is " ever open; and amply will it repay the most attentive and diligent research." The contents are somewhat miscellaneous, incomplete in themselves, and more or less unconnected, having been collected mainly from scattered papers published in the London Med. Gazette. The work is divided into two parts; the first of which contains some Introductory Remarks, "General Remarks on the Treatment of Inflammation," "Chemosis," "Remedies employed in the Treatment of Inflammation," "Strumous Inflammation of the Conjunctiva," "Morbid Conditions of the Conjunctiva and Sclerotic," "Irritable Ophthalmia," "Purulent Ophthalmia," "Injuries of the Conjunctiva," "Opacities of the Cornea," "Pterygium," "Malignant Diseases," "Cataract," "Strabismus," "Fistula Lachrymalis," "Iritis," "Myopia," "Presbyopia," and "The Selection of Spectacles." The second part contains—"Remarks on Fistula, Compound Fractures of the Cranium, Hernia, Treatment of Bronchocele, Cancer of the Breast, Suppression of Urine, Uterine Hæmorrhage, Gout and Rheumatism, Diet and the Disorders of Digestion."

The author's introductory remarks are, as far as they go, judicious. It is true that, "by careful observation, an accurate knowledge is obtained of the symptoms, nature, and terminations, as well as of the degrees, and various forms of morbid action, which arise in each particular structure. But the knowledge thus acquired is not limited to diseased conditions of the eye, it can be used most advantageously in assisting the diagnosis of diseases in other organs, where a like structure exists, but which are concealed from view; and, lastly, by seeing the effect of remedies used in the cure of various morbid conditions of the eye, much practical and highly important information, relative to the treatment of diseases in other parts of the body, may be obtained."

In his remarks on the Treatment of Inflammation, Dr. Hall directs attention to the necessity of removing the exciting cause if it be still present. He briefly mentions a case where an acute attack of iritis was induced by a blow from a stick in passing through a thorn fence. At thc time of the patient's application he had been treated by purging and bleeding—in addition bleeding, with calomel and opium were directed, which checked without curing the disease. After several days, on minute inspection, the smallest portion of thorn was found sticking in the cornea:—this was removed and the man recovered. This case, with several others fresh in our recollection, prove the necessity of careful attention to the history of the case, its peculiar features, and a careful examination of the eye in all doubtful instances.

"The same disease," says Dr. Hall, "in all cases does not require similar treatment." Our own experience would induce us to say that we must be guided in every case not only by the age, sex, occupations, and peculiarities of the patient, but by the characters, both general and local, which the disease may present. On no account should the diseases of the eye be differently treated from analogous affections in other parts of the body, merely because they are seated in the organ of vision, but influenced by the same considerations, and using as large a proportion of common sense, it is as necessary to remove complications, and to study its peculiar features. In chronic cases a mere change of treatment is often sufficient to produce a cure, where an opposite plan has been pursued for some time unsuccessfully—such as the substitution of soothing measures, or, in suitable cases, of tonics or nutritious diet, for antiphlogistic treatment and mercury.

*Some of the more Important Remedies in Ocular Inflammation.*

Under this heading Dr. Hall discusses the advantages and propriety of bleeding, purgatives, mercury, nauseants, tonics, counter-irritation, local applications, stimulants and escharotics. We are told that a knowledge of the due application of bloodletting can only be acquired by careful observation and extensive practice. Blood should, if possible, be taken from the bend of the arm, since opening the external jugular vein, or the temporal artery, present disadvantages, without any corresponding benefit. A large opening is recommended to be made in the vein, and in all acute cases, one very large general bleeding in preference to several small ones. Repetitions are rarely required, but leeches may be applied around the eye, but not on the lids, as they are apt to occasion swelling and ecchymosis in that situation. Purgatives are often required to correct the deranged condition of the alimentary canal, or to reduce the plenitude of the system. We have found an active purge, which both clears out the alimentary contents and promotes the flow of the biliary and intestinal secretions, most highly valuable in acute conjunctival inflammation, whilst mild and continued purgation, with or without tonics, is beneficial in several chronic inflammations, especially connected with deranged and depressed health. Dr. Hall cautions against the abuse of these medicines, for however necessary they may be in certain cases, their employment in this country is by far too common, and the doses in which they are given absurdly large, "that the evils arising from the abuse of aperients are too often overlooked," and he feels certain "that many who have commenced their practice with the idea that large doses were required in almost every case, more or less alter their opinion as it becomes matured by time and experience."

Dr. Hall, in his comments on mercury, agrees with Dr. Holland in respect to calomel, "that its combination with purgatives in these cases both obscures and impairs its effects,"* and that therefore, as a general rule, it should be given alone. Where a mercurial is to be continued for some time, Dr. H. also agrees with Dr. Holland in thinking that no medicine or combination of medicines has an equally beneficial effect as the bichloride. He has seen the advantages of a long course in chronic iritis, and also in several cases of paraplegia—the slow progress of the disease giving full scope for its effects. For ourselves we prefer the blue-pill in combination with conium, which we have found more safe and certain, and at the same time milder in its operation.

Dr. Hall states that to Mr. Middlemore of Birmingham is due the credit of directing attention to the utility of sulphate of quinine in certain strumous inflammatory conditions of the eye, particularly of the iris and aqueous humour. Ophthalmic medicine is deeply indebted to Mr. Middlemore for many practical suggestions, and for unwearied and untiring observation. The benefit of tonic treatment in the majority of the

* Medical Notes and Reflections, p. 242.

strumous diseases of the eye is now fully allowed in Great Britain and Ireland, and we find Dr. Erdman, in Graefe and Walther's Journal, dwelling at some length on the excellent effects of quinine in some of the most obstinate cases of strumous ophthalmia.* Dr. Hall narrates the case of a child, in illustration, who was brought to him in 1842. She was remarkably fair and handsome, and had already suffered from "scrofulous inflammation of both eyes" twelve months. After quinine had been taken for a month or six weeks, attention being also paid to diet and exercise, so great a change was wrought in her personal appearance, that it was almost impossible to recognise the puny, cross, feeble, delicate, little creature that was first brought, in the robust, rosy-cheeked child then standing before him.

It has been our lot to find, in a somewhat extended experience in the treatment of ophthalmic disease, that tonics and good diet are occasionally needed in almost every form of inflammation which affects the eye. We have found passive cases in every ophthalmia from the most obvious and simple variety of conjunctivitis up to the most obscure type of retinitis—cases which have rapidly improved under nutritious aliment and tonics, but which have become aggravated and have resisted opposite measures for an indefinite period. Our rule, from such experience, is never to judge beforehand what measure we shall institute for any individual case, but to be guided entirely by its special indications, although when we speak generally of a disease, we may say that such and such treatment is required.

Of counter-irritations Dr. Hall remarks, that "too much care cannot be exercised in daily watching their effects when applied to the head and face." In our own practice, we never employ any form of counter-irritation which is likely to have any permanent mark in a situation exposed to observation—especially the face—or any mode occasioning much suppuration in patients whose constitutional powers are not able to bear the discharge.

Stimulants and escharotics "very often increase internal and deep-seated inflammatory affections of the eye when injudiciously employed, however useful under proper management," and, we would add, not unfrequently augment or produce superficial diseases, which they were intended to cure. Dr. Hocken † has drawn attention to the subject of granular conjunctiva, which he has shewn to be produced in more cases than are suspected, solely by the unscientific, indiscriminate, and repeated use of the nitrate of silver, either in substance, or as a strong solution.

### STRUMOUS INFLAMMATION OF THE CONJUNCTIVA.

Dr. Hall states, that "on exposure of the globe to view, the ocular conjunctiva is found with its vessels injected with red blood, as in a case of simple ophthalmia; the palpebral portion of the membrane has its vessels

---

* Vide the July Number of this Journal for 1842, p. 189.
† Prov. Med. and Surg. Journ. vol. I. 1841-2, p. 390.

also more numerously distended with the red fluid. In those instances in which the palpebræ are found tumid and red, ulceration of the cornea will also be discovered." The fact is, we believe, that strumous conjunctivitis presents many varieties, since any of the ordinary forms of conjunctivitis may be modified by the strumous constitution, but that these do not strictly come within the meaning of the term "strumous conjunctivitis," which should be confined to those cases where intolerance of light and the formation of phlyctenulæ constitute the more prominent symptoms, being unattended by much redness, at all events during its early stages. Out of fifty patients under Dr. Hall's care with various affections of the eye, no less than thirty-six had scrofulous ophthalmia. "Many of these cases occurred either in young females with suppressed or irregular uterine action, or in children near the period when the generative powers are becoming developed." This does not accord with our own experience, having almost invariably met with the disease during primary or secondary teething, so that it is probable that Dr. Hall and ourselves are alluding to distinct affections.

Following in the well-beaten path, Dr. Hall attributes the intolerance of light, so marked in this disease, to "mordid sensibility of the retina." We are inclined, however, to believe, with Dr. Hocken, "that the true retina has nothing to do with its production.* This gentleman has proved to our satisfaction that the essential nature of the disease consists in a morbid sensibility of the terminal filaments of the ophthalmic division of the nervous trigeminus, propagated through the great sympathetic.†

A very important part of the subject relates to treatment. Dr. Hall recommends counter-irritation, by means of small blisters behind the ears or at the back of the neck, tonics, and mild applications to the eye, with especial attention to important functional disturbances. "Where the disease is accompanied by profuse perspiration," he remarks "or diarrhœa, blisters must be employed with very great caution, the slightest counter-irritation frequently sets up an inflammatory action, which ends in gangrene and mortification." Attention is also directed to diet, air, exercise, and clothing.

Dr. Hall does not allude to a plan of treatment strongly recommended by Dr. Hocken in the paper to which we have previously referred. This latter gentleman states, that the application of the nitrate of silver to the *outside* of the lids is so successful in strumous (and some other forms of) conjunctivitis, that he has never known it fail in arresting the disease *immediately*, in recent cases, when combined with appropriate constitutional measures, or after two or three applications in the more chronic. The patient's eyelids being closed, and put slightly on the stretch, a clean piece of the stick of nitrate of silver (previously moistened) is to be passed very lightly and smoothly over the surface of the skin of the upper and lower eyelids, bringing the sides and not the point of the stick in contact with the skin. The object of this application, says Dr. Hocken, is only to blacken and not produce any severer effects, and it will be found that,

* Lancet, vol. I. 1842-3, p. 258.

as soon as the remedy has had time to produce its peculiar action, it will completely relieve the intolerance of light, the lachrymation, and, what is of more importance, the spasmodic strivings of the orbicular muscle, by which the patient is rescued from that constant irritation and pressure which maintains and aggravates the affection.

The further treatment of the attack, consists in administering quinine with a mercurial in moderate doses, once or twice, and following it up by an aperient dose of rhubarb, ginger, and sulphate of potash.

This plan of treatment has been tried and approved by many competent judges, amongst whom we may mention the names of Mr De la Garde, (senior surgeon to the West of England Eye Infirmary,) and Dr. Lanyon.* Its recommendation also drew forth a paper from Dr. Furnivall, physician to the General Infirmary, Hertford, in praise of the tincture of iodine similarly applied. He remarks, that " having been for many years in the habit of prescribing for cases of conjunctivitis, (chiefly strumous, and therefore usually the most obstinate,) and having enjoyed many opportunities of watching these distressing cases amongst the numerous out patients of the Infirmary at this place, I would beg to recommend to Dr. Hocken a trial of painting the palpebræ of the affected eye or eyes with the alcoholic solution of iodine, commonly called Træ. Iod. For very fine-skinned children the strength may be lowered."† Dr. Furnivall was led to apply this remedy by reflecting on the continuity of surface between the skin and conjunctiva, "and, truly," he remarks, " the effects have been beneficial in an extreme degree." The distressing lachrymation is immediately relieved, and he has had reason to think that in several cases he has stopped ulceration of the cornea. The remedy is easily applied; it dries on; gives no trouble, and requires some two or three applications every week, according to its effects.

Nothing worthy of extract is contained in the section on " morbid conditions of the conjunctiva and sclerotic." Two or three cases are given in illustration, one of which was of the gonorrhœal type. We have met with the disease more commonly in persons somewhat more advanced in life than would appear to be the case with Dr. Hall, and very often combined with more or less inflammation of the iris.

### IRRITABLE OPHTHALMIA OF FEMALES WHEN SUCKLING.

Dr. Hall remarks, with justice, that this form of ophthalmia is rather to be regarded as a distinct type of irritable conjunctivitis, than as a distinct and separate disease, just as strumous ophthalmia is only a peculiar form of several strumous diseases of the eye. We are indebted to Mr. Middlemore for calling the attention of the profession to this form of the disease, and although, from our own experience, we should say that it was not very uncommon, it has not been mentioned in the works of Lawrence, Mackenzie, or Tyrrell.

" The disease," says Dr. Hall, " attacks women when suckling, particularly

---

* Vide Lancet, Jan. 21st, 1843.

† Lancet, vol. I. 1842-3, p. 405.

if they have continued to nurse their children too long, and more especially occurs after this plan has been adopted two or three times. It is a very common opinion, that suckling prevents impregnation; women among the lower classes, supposing that conception cannot again take place until the child is weaned, they therefore continue to nurse their infants for a considerable period, and this frequently induces an attack of amaurosis or this peculiar form of ophthalmia." 33.

The fact of its frequently preceding or accompanying an atonic form of amaurosis, gives a degree of importance to the disease, without which it would scarcely deserve a separate description. Professor Nasse * has also described a very dangerous form of corneitis, which commences with the irritable form of conjunctivitis. The conjunctival inflammation rapidly passes to the cornea, and is accompanied by darting pains in the eye and orbit. From the third to the eighth day an abscess forms between the layers of the cornea, when the inflammation diminishes, but the onyx generally makes its way into the anterior chamber. The principal part of the treatment consists in separating the child from its mother, and in the administration of tonics.

There can be no doubt that these pathological states, which come on from undue or excessive lactation, depend on the depressed and irritable state of constitution which is thus induced—the general symptoms in each being those of debility and hectic. Hence, Dr. Hall is perfectly correct in stating that " the first indication is the removal of the exciting cause." " At once," he says, " let an extinguisher be put upon the cause of the disease; from that moment insist on the weaning of the child." As soon as the exciting cause is removed we may generally conduct the disease to a successful termination, if seen sufficiently early. Dr. Hall states, that he is acquainted with a lady who is always attacked with irritable conjunctivitis when suckling her children; and which, when he last saw her, had continued for a considerable period, but on advising her to wean her baby, the eye was quickly restored to a healthy condition.

Mr. Middlemore met with a curious fact in three females, who had continued to suckle for an injudiciously long period during the year 1836. One eye only was affected in each case, and on enquiry he found that the subjects of the malady had suckled only with the breast of the side corresponding to the diseased eye.†

### Purulent Ophthalmia.

Dr. Hall is fully convinced that any inflammation of the conjunctiva, attended with a purulent discharge, although described as presenting different aspects, is nevertheless the same, although its degree of severity may differ. No doubt, the pathology is essentially similar in the common purulent, the gonorrhœal, and the infantile purulent ophthalmia, yet, in a

* Ammon's Monatschrift fur Medicin.
† Trans. Provin. Med. and Surg. Assoc. vol. V. p. 372.

practical point of view, there are differences sufficiently great to warrant their separation. Dr. Hall's section on the history of the disease is a mere condensation from the valuable work of Mr. Middlemore.

"Purulent ophthalmia," says Dr. Hall, "in the adult, for the most part in this country arises from the contact of purulent secretion." This statement we believe to be incorrect, both in the common and gonorrhœal forms. It is obvious that isolated cases, which arise without any connexion with diseased persons, must originate from some other cause than contact, and where the disease prevails extensively, it is where many are crowded together in a confined space—the poison acting through the medium of a confined atmosphere on such predisposed individuals as are exposed for a sufficient length of time to its influence. Mueller has come to this conclusion from a very extensive experience. He has never seen it in the friends of patients who were allowed to visit the sick—such visits being limited to half an hour, and taking place in a separate apartment, whilst the nurses and medical attendants, &c. were among the sufferers.* There can be no doubt that both the common and specific forms may arise from the contact of matter, but these are rather the exceptions than the law. M. Carron du Villards states, that the inoculated disease affects one eye, and he has observed that, in left-handed individuals, it was the left eye which was affected, and vice versa. (Guide Pratique, vol. II. p. 542.) Dr. Hall mentions that he once had an attack of the disease himself, from the accidental squirting of gonorrhœal matter into his eye. "A patient," says he, "consulted me for a discharge from the urethra: I advised the use of an injection, and on throwing some warm water up the penis with a syringe, a portion went into my right eye. Considerable pain was felt, immediately followed by inflammation, and the next day by a slight purulent discharge. M. Decondé's valuable experiments on the prevention of purulent ophthalmia are not alluded to by Dr. Hall. M. Decondé's conclusions are—1st, that chlorine and chlorurets are certain disinfectants for gonorrhœal and ophthalmic contagions, and that they are to be preferred to all others;—2nd, that, to preserve soldiers free from ophthalmic infection, it is not enough to use lotions, but that the atmosphere itself should have chlorine suspended in it;—3rd, that medical attendants who finger affected eyes should dip them in chlorine to prevent the carrying of virulent matter from one eye to another, or from one person to another, and that the same recommendation must be given to those who handle parts affected with gonorrhœa.†

In the consideration of "Chemosis," Dr. Hall adopts the theory of Messrs. Travers, Middlemore, and Tyrrell, as regards the mechanical obstruction of the corneal circulation, and advises the performance of the radiating incisions recommended by the latter gentleman. Dr. Hall remarks, "on reading over what I have written on the division of the chemosed parts, I think I have hardly done sufficient justice to the claims of Mr. Middlemore, who certainly was the first to point out the advantages arising from free division of the strangulated parts." The theory is not

* Erfahrungssätze, p. 80-2.

† L'Examinateur Medicale, October 24th, 1841.

new on the part of Mr. Tyrrell, since we find the doctrine distinctly laid down in the Synopsis of Mr. Travers (but without any practical inference), in the Jacksonian prize Essay for 1831, by Mr. Middlemore; the London Medical Gazette for 1832-3, in Mr. M.'s Lectures, and in numerous parts of the first volume of his "Treatise," published in 1834. Speaking of the results of purulent ophthalmia, we find Mr. Middlemore, at p. 124, vol. i, stating that, "when sloughing of the cornea takes place in the progress of purulent ophthalmia it is not generally from the occurrence of inflammation of that tunic, but from the interruption to its nutrition, owing to the compression caused by the chemosis. Severe and prolonged chemosis causes mortification in the case in question, as I apprehend, because it generally takes place where such chemosis exists in its severest form. Sloughing of the cornea is more frequently associated with chemosis than with any other affection of the organ of vision. And it may be mentioned, in additional evidence of the truth of this position, that in all such instances the mortification begins in its superficial layers." "Mr. Tyrrell," says Mr. Middlemore, in a note quoted by Dr. Hall, "can fairly lay claim to the radiated incisions, but whether they are in all instances safe and practicable, and whether they are better than the curved or semilunar ones, which stop short of the greatest diameter of the eye, are questions I cannot, as a *practical* man, answer in the affirmative." 57.

### Malignant Affections.

Under the head of "Malignant Disease," Dr. Hall narrates the case of Wm. Bartrup, æt. 9, who was first brought to him when seven years old. "The eyelashes of the left eye were gone, and the lower lid partially everted, the globe of the eye was much increased in size, and the vitreous body turbid, and of a dirty brown colour." The mother stated that, some months before, the pupil had a yellow appearance, which increased slowly at first, then quickly, when the eye began to be bloodshot. A mild mercurial plan of treatment was commenced, and steadily followed for more than six months, with great attention to diet. At the end of four months some slight improvement was evident, and after six, the diseased mass gradually began to subside. His general health, also, was "quite robust." The disease, however, made great progress in the course of a year, and mercury, which hitherto seemed to possess the power of arresting it, appeared now quite inert. The eye-ball increased daily in size, but the boy complained of no pain.

Dr. Hall eventually extirpated the globe "in the usual manner." "The boy in this case got quite well over the operation, and is now quite recovered. The other eye is much improved, and the mother told me, a few days ago, that he was 'in the best of health.'" 74.

The question is—was this a case of genuine "malignant disease?" for, if so, an example where extirpation proved so successful at such an advanced stage, would be most encouraging and valuable. To us, however, it appears to present no one indication or symptom of a decidedly malignant character, and we are sorry to say that, in our opinion, there is no encouragement to perform the operation in cases of confirmed and genuine fungus hæmatodes, which we must still consider as hopeless.

"I shall only offer a few remarks on cataract," says Dr. Hall, "in this place, because I hope at no very distant period to devote a greater space than the limits of the present volume will permit to its consideration." The "remarks" which are given do not contain anything but what was previously well-known. He narrates two cases of partial cataract which had been mistaken for amaurosis. "They point out the absolute necessity of dilating the eye (iris ?) with belladonna in all cases of suspected cataract, before a decided opinion is pronounced." 79.

We find the author lauding the iodide of potassium as an adjuvant in the treatment of acute iritis. It is, he states, a very valuable remedy, and next to mercury the best we can employ. "In every case I am called upon to attend," says Dr. Hall, "I give small doses of the above (℞. Iodidi potass. gr. ij. ad iv, Syrup. aurantii ʒj, Aquæ rosæ ʒix. M. ft. haustus ter in die sumend.) in addition to the calomel and opium, and with the greatest possible success." Dr. Hall narrates a case in which he employed this remedy in combination with bleeding, calomel and opium, strong mercurial ointment and opium, and the extract of belladonna—free purging having been previously employed. "This treatment," he remarks, "produced the most happy effects." We might enquire what share the iodide of potassium and orange syrup had in producing these beneficial results, when combined with such powerful auxiliaries? Can Dr. Hall answer the question ? for, to say the truth, we cannot. Never having found the need of such an adjuvant, we should be inclined to doubt its value, and should at all events spare the patient the infliction of swallowing three unnecessary draughts a day—unless, perhaps, we put up and charged for the medicine.

Dr. Hall goes on to offer some common-place remarks on iritis from wounds, with a case in illustration, and on the syphilitic, scrofulous, and arthritic forms of the disease—recommending colchicum in the latter. We have often seen it employed in cases called "rheumatic" and "gouty," but with little or no benefit. The first part of the work is concluded by imperfect sketches of myopia and presbyopia, and a section on the "selection of spectacles."

"Lastly," says Dr. Hall, "let me urge the importance of having glasses of good material, and accurately ground, in order that the refraction may be as perfect as possible, and the power of both glasses the same. Much knowledge is required to accomplish this; and if good glasses are required, some respectable optician must be consulted. Advertising opticians of every grade and class, from the highest to the lowest, are not to be depended upon, and I advise both my readers and patients requiring spectacles, as they value the blessings of sight, to avoid them. 'Non est tibi fidendum, ut qui toties fefelleris.'" 125.

### FISTULA IN ANO.

"*Manner in which the Operation is performed in Paris.*—M. Roux, of the Hôtel Dieu, Paris, performs this operation in a different manner to that performed by Pott, and followed by English surgeons. He introduces a long piece of boxwood into the rectum, having its concavity towards the fistula. A silver director is then introduced along the fistulous track, and its end made to come in contact with the wooden gorget in the bowel. A long, strong, narrow sharp-pointed knife is then introduced along it till it comes in contact with the piece of box-wood. The director is then withdrawn; and by keeping the point of the knife fixed upon the gorget, and withdrawing both together, all the parts between the fistula and rectum are divided. This part of the operation completed, the bistoury is exchanged for a scalpel, and all the hardened base is carefully dissected out. A thick, long probe is then procured, having a button at one end; this is covered with charpie, smeared over with some yellow-looking ointment, and the wound crammed full of it to the bottom." 139.

Dr. Hall thinks that three objections may fairly be raised against this operation—1st. That with the wooden gorget we are operating in the dark, with nothing to guide us.—2nd. That the dissecting out of the hardened integument, which is performed "by two incisions crossing each other at right angles, the flaps being each lifted up with a pair of forceps, and cut out with the knife," is worthy only of a by-gone day, but not of the nineteenth century, being only calculated to add to the sufferings of the patient.—3rd. That the plan of stuffing the wound daily with charpie, is attended with great pain, produces considerable uneasiness, and most certainly impedes the cure. "It is chiefly," says Sir B. C. Brodie, "in consequence of the use of too much lint in dressing, that further operations are so frequently required before the cure is completed."

It is absolutely necessary to substitute the ligature in those patients on whom no operation can be performed without danger from severe hæmorrhage.

A section follows on Compound Fracture of the Cranium with Depression, in which several cases are given to prove that these injuries frequently do well under ordinary treatment, without the use of the trephine. The results of an experience of some years, induces him to conclude that, in the great majority of cases of compound fracture of the skull with depression, we ought not to trephine, unless it appears clear that the brain is suffering from pressure. If, however, the bone be much comminuted, and the wound in the integuments large, "some of the pieces may be picked from the brain, and others elevated, the several splinters sticking in the dura mater removed, and this without additional injury to the scalp," even in the absence of urgent symptoms.

Omitting the chapter devoted to the consideration of an "Operation in a Case of very large Strangulated Hernia," we may pass on to the Treatment of Bronchocele. The chapter concludes with the following remarks.

"1st. That although it abounds in certain localities, we know not on what it depends, or why it should abound more in Switzerland or Derbyshire than other places. 2nd. That we have no reason for concluding that goitre should produce cretinism, although the two are frequently combined. 3rd. That it is highly important to attend to the general state of the secretions before attempting to make use of specific remedies, and also that considerable advantage appears (in the cases I have seen) to result from fomenting the part affected with warm water, (previous to using the iodine ointment); the application of blisters, and the local abstraction of blood by leeches; the exhibition of liquor potassæ, and alterative medicines." 177.

In his remarks on Cancer of the Breast, Dr. Hall thinks, that too much attention cannot be paid to the state of the system, both before and after the operation, and that the disease returns in some instances from a want of these precautions. The sooner we perform the operation the better, for delays are dangerous: and he would rather run the risk in very rare instances of removing a non-cancerous breast, than by not removing it expose his patient to the chance of becoming a victim to this relentless disease. No prudent surgeon would operate in cases where the whole of the disease could not be removed, or where there is reason to suppose that internal organs are affected. Mr. Liston states that, when enlarged glands are perceptible above the clavicle, or in the intercostal spaces, the practitioner who would advise interference with the original tumour must be grossly ignorant, atrociously unprincipled, or of unsound mind. Where dyspnœa is present, Sir A. Cooper found, on post-mortem examination, water in the chest and tubercles on the pleura. An operation is advised in advanced cases by Dr. Hall, "not because the disease does not frequently return, but because it affords the only chance." He thinks it our duty to tell the patient how very faint our prospects of success are, and yet that cases by Sir A. Cooper and others are recorded which, had they been permitted to run their course, must in all probability have terminated fatally in a few weeks, where an operation had restored the patients to comparative health, and even added some years to their existence.

The general treatment of these cases may be summed up in the words of Dr. Copland—to support "the energies of the digestive functions and the abdominal secretions and excretions, and to impart vigour to the frame by suitable diet and regimen," equally avoiding stimulation on the one hand, or depression on the other; "for if you do," says Sir A. Cooper, "it will be the sure way to hasten the progress of the disease."*

### SUPPRESSION OF URINE.

This disease is described as one of rather rare occurrence. Dr. Elliotson had only seen one case, and that occurred after the patient had taken a quantity of corrosive sublimate by mistake. A diminution of the natural quantity of urine occurs in fevers, small-pox, after accidents and surgical operations, from injuries or disease of the spinal cord, and in cases of malignant cholera—but the secreting office of the kidneys may be completely suspended, independent of acute disease, and quite independent of any detectable alteration in the structure of these glands. All such cases end in coma; some with, and others without convulsions—but all have evident symptoms of apoplexy. Suppression of urine includes therefore two divisions, the partial and the complete.

Partial forms are far from unfrequent. Sir B. C. Brodie, in a note to Dr. Hall, states that, in the great majority of cases of this kind that he

* Lectures on Surgery, p. 343.

has seen, there has been some obstruction to the flow of urine; and it is curious that a calculus blocking up *one* ureter, or a tumour pressing on *one* ureter, will sometimes stop the secretion of urine in *both* kidneys. In one case there was a very enlarged prostate, which probably closed the orifices of the ureters, but the body was not examined after death. In another case there was a medullary fungus of the mucous membrane of the bladder producing this effect. In a third, an enlarged prostate prevented the patient from emptying his bladder. For some time he had not secreted more than half a pint of urine daily, but it was trebled immediately on the catheter being used two or three times daily.

The great and urgent danger of complete suppression were first made known by Sir H. Halford, who narrates an interesting case in the 6th volume of the Medical Transactions. These cases commence very insidiously.

"You find a man," says Dr. Hall, "following his usual occupations, he sends for you to draw off his water, and you discover none in the bladder on the introduction of the catheter. There is a dull uneasy sensation about the loins; a feeling of oppression at the pit of the stomach; a disinclination to move from place to place; a loathing of food. These symptoms are followed by rigors, pain in the head, and drowsiness, ending in coma and death." 192.

The cause may be either functional or organic. Dr. H. attended a case of organic disease of the kidney at Kensington, in which no urine was secreted for the last week of existence. Dr. Hall's attention was however "more particularly directed to this complaint by the following interesting case, in which there was for many hours a total suppression of urine, yet the man recovered, and is now enabled to attend to the duties of his station."

Joseph Lambert, ætat. 27, rather below the middle height, thick-set and stout, with a short neck, and florid complexion; is often exposed to cold and wet in his capacity of under-gamekeeper to Earl Spencer. March 18th, 1840, he called, complaining of pain at the pit of the stomach, and diarrhœa. 19th. Dr. H. called to see him at 9 in the morning, and found him walking about in great pain; his face flushed; tongue coated; pulse 120, quick and full. At 7, feeling uneasy, he had got up to make water, and could only void a few drops—has not passed any urine for the last 18 or 20 hours—upon placing the hand above the pubes it was at once evident that the bladder was not distended; a tea-spoonful of very acid urine (all he had passed) was shown. Not a drop of water escaped on the introduction of the catheter. The pain in the loins was at times severe, followed by a sensation "as though the small of his back was half broken." The eye was dull and heavy, the pupils considerably dilated, and he complained of slight drowsiness and pain in the head. V.S. ad ℥xvi. The blood was dark and thick; since the bleeding, has had a motion, which is pale and watery. He was ordered to drink freely of linseed tea, to put his feet in warm water, to be followed by mustard poultices; to have a warm bath, and not to leave his bed. ℞. Hyd. chorid. gr. viii.; Pulv. lyttæ, gr. j.; Ol. tiglii, ♏iv.; Ext. hyosciami, gr. iv.; Misce, et divid. in pil. iv. Sumat j. quaque tertiâ horâ. ℞. Sodæ sesquicarb. ʒj.; Pulv. potassæ, nit. ℈j.; Træ hyosciami, ʒij.; Tr. scillæ, ʒj.; Tr. lyttæ,

ʒj.; Mist. camph. ℥vss. M. capiat coch. larga duo quaque 3tia horâ. ℞. Ol. terebinth. ℥ss.; Spir. camph. ℥j.; Linim. sapon. ℥iss. M. ft. embrocatio lumbis applic. Three o'clock, p.m., a blister to the loins.

6, P.M. Pupils much dilated; complains of more pain in the head; answers questions in a quick, sharp manner, and is evidently becoming delirious. 12, P.M. No better; has had two more evacuations, but has not passed any water. 20th. 9, A.M. Has passed a bad night. Face flushed; pupils still dilated; complains of great thirst; skin hot and dry; pulse 90, slow, and labouring. The catheter was introduced, and two ounces of urine drawn off—all that had been voided for 42 hours. Some James's powder with calomel was now ordered, and spir. junip., træ humuli, and magnes. sulph. as a draught. At 9 o'clock, P.M. he was decidedly better, the skin moist, pulse 72. Had made about three ounces of urine since the morning. ℞. Pulv. antim. pot. tart. gr. j.; Magnes. sulph. ℥ij.; Træ lyttæ, ʒj.; Syrup. tolut. ℥ss.; Aquæ, ℥vss. M. sumat. coch. amp. duo quaque 2nda horâ. 21st. The only food he has hitherto taken is linseed tea. This morning he is much better; less pain in the head; no pain in the loins; skin moist; pulse 82. Bowels well opened during the night, and the secretion of bile increased. To continue mixture. Has passed since last night about ten ounces of urine. From this time he daily improved, and was soon able to return to his usual duties.

The chapter which succeeds contains the particulars of two or three cases of uterine hæmorrhage, where the placenta was situated over the os uteri.

Twenty pages are devoted to the consideration of gout and rheumatism, containing no addition to our knowledge of these diseases, nor of the best modes of treating them. Dr. Hall thinks that colchicum must be continued for some time after the cure of an attack of gout, if we wish to remove the diathesis altogether, "and," says he, "I honestly think that this medicine may be made a preventive as well as a curative of gout." The chapter is concluded in the following language:—"As I write my memory recals cases of *bloated dropsy, of livid asthma struggling for breath, of tottering palsy, of yellow-faced jaundice, of red-eyed delirium, of limping gout grinning with pain, of musing melancholy,* and *incurable insanity;* and if a pupil ask me the causes of these fearful inflictions, I answer, they are too often the diseases of intemperance!!" "Temperance," says Burton, "is a bridle of gold, and he who can use it aright is liker a god than a man." "If you would be well," says Abernethy, "live on sixpence a day and earn it." "The pith," says Dr. James Johnson, "of all that has been written on hygiene, and the prevention of disease—and of the Proteian disorder among the rest—might be included under two heads, almost in two words—TEMPERANCE and EXERCISE—we must keep the body active and the stomach empty."

The work is concluded with some judicious remarks on diet and the disorders of digestion. "The age of farce," Dr. H. remarks, "is almost gone; let us hope those also of the St. John Long's, of the Balm of Gilead, the Real Blessing to Mothers, Morrison's and Parr's Pills, are also numbered, and that, for the future, we shall be permitted to live without groundless apprehension, till we reach the winter of old age, and at last die a natural and peaceful death." We fear that a superstitious love and belief in mystery is a predominant feature of the public mind, and will still cast a charm around any novelty which promises more than genuine knowledge can hold out.

In stating our opinion of this volume, we find that it is utterly void of original matter, and consists of incomplete and unconnected materials; the first trait renders it of but little value to the experienced practitioner, whilst the last renders it equally useless to the student. The first part is clearly written, and shows that the author is tolerably well acquainted with ophthalmic pathology. The chapter on suppression of urine is the best of the second part, and is worthy of perusal, but, on the whole, we know not to what class of readers we can conscientiously recommend the volume.

